# Symptomatic Trifascicular Block in Steinert's Disease: Is It Too Soon for a Pacemaker?

**DOI:** 10.1155/2016/6372181

**Published:** 2016-02-28

**Authors:** Glenmore Lasam, Roberto Roberti, Gina LaCapra, Roberto Ramirez

**Affiliations:** ^1^Department of Internal Medicine, Overlook Medical Center, Summit, NJ 07901, USA; ^2^Section of Cardiology, Overlook Medical Center, Summit, NJ 07901, USA

## Abstract

We report a case of a 62-year-old male with Steinert's disease who presented with progressive intermittent episodes of lightheadedness five years after he was diagnosed with the disease. On evaluation, he developed a new onset trifascicular block (first degree atrioventricular block, new onset right bundle branch block, and left anterior fascicular block). A dual chamber pacemaker was inserted and lightheadedness improved significantly.

## 1. Introduction

Steinert's disease or myotonic dystrophy type 1 may present as symptomatic trifascicular block which may progress to high degree atrioventricular block and fatal arrhythmias that could lead to sudden cardiac death. This could pose a dilemma to the clinician whether to do conservative surveillance or immediate pacemaker insertion to abate symptom.

## 2. Case Presentation

**Figure 1:**
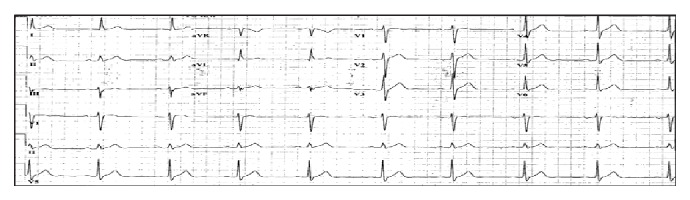
Patient's baseline 12-lead electrocardiogram. The tracing revealed sinus bradycardia (with heart rate in the 55) with normal axis.

**Figure 2:**
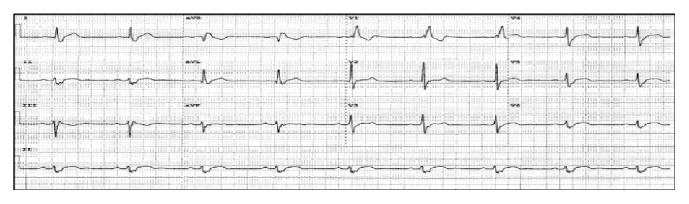
Patient's 12-lead electrocardiogram when he presented with frequent lightheadedness. The tracing revealed sinus bradycardia with first degree atrioventricular block, right bundle branch block, and left axis deviation consistent with left anterior fascicular block (new finding).

A 62-year-old male was diagnosed with Steinert's disease (myotonic dystrophy type 1) at the age of 50 when he presented with bilateral leg muscle weakness associated with numbness in his toes and occasional dysphagia compatible with esophageal dysmotility. The diagnosis was confirmed by genetic testing and electromyography. His sister, nephew, and niece were diagnosed with the same disease. Five years ago, he gradually developed jaw weakness and started complaining of intermittent episodes of lightheadedness and easy fatigability. He denied any syncope, chest pain, dyspnea, orthopnea, or palpitations. He had trace bipedal edema and 4/5 bilateral lower extremity motor strength. Electrocardiogram showed normal sinus rhythm, first degree atrioventricular block, left axis deviation, new onset right bundle branch block, and left anterior fascicular block (trifascicular block) which were new findings (Figure 2) compared to five months earlier (Figure 1). Holter monitoring did not demonstrate any pauses. Transthoracic echocardiogram revealed mild left ventricular hypertrophy with normal ejection fraction. Adenosine myocardial perfusion imaging showed moderate distal anterior and distal lateral ischemia with ejection fraction of 53%. He was started on aspirin.

**Figure 3:**
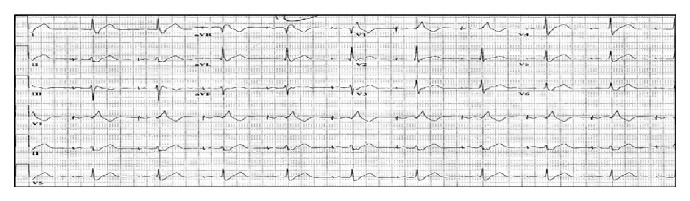
Patient's 12-lead electrocardiogram after the pacemaker insertion. The tracing revealed electronic atrial paced rhythm, left axis deviation, and right bundle branch block.

Three months after the onset of the trifascicular block, the lightheadedness became more frequent and, even though a repeat electrocardiogram revealed no changes, a dual chamber pacemaker was recommended due to the unstable progression of conduction disease in myotonic dystrophy. The pacemaker was successfully inserted as confirmed by electrocardiogram (Figure 3) and subsequently the lightheadedness improved.

## 3. Discussion

Myotonic dystrophy (DM) is an autosomal dominant disorder characterized by myotonia (delayed muscle relaxation after contraction), weakness and atrophy of skeletal muscles, and systemic manifestations including endocrine abnormalities, cataracts, cognitive impairment, and cardiac involvement [1]. There are two types of myotonic dystrophy. Classical DM, called Steinert's disease or myotonic dystrophy type 1 (DM1), has been associated with the presence of an abnormal expansion of a CTG trinucleotide repeat on chromosome 19q13.3 [2] in the* DMPK* gene that codes for myotonic dystrophy protein kinase, a protein mainly expressed in smooth, cardiac, and skeletal muscle cells [3]. Myotonic dystrophy type 2 (DM2) is caused by a dominantly transmitted CCTG repeat expansion in intron 1 of the zinc finger protein 9 (ZNF9) gene on chromosome 3q [4]. A critical element in the pathogenesis of this disease in both types is the intranuclear accumulation of the expanded RNA sequences which disrupt the regulation of alternative splicing of mRNA and perturb the expression of many genes; thus multiple systems are affected clinically. DM1 is more common, affecting approximately 1 in 8000, making it the most common adult form of muscular dystrophy while DM2 is less common affecting approximately 1 in 20000 [5].

Sixty-five percent of DM1 patients have an abnormal ECG in which conduction abnormalities are the result of myocyte hypertrophy, fibrosis, focal fatty infiltration, and also lymphocytic infiltration, which can occur anywhere along the conduction system including the His-Purkinje system [6]. Prolongation of the PR segment occurs in roughly 20–40% of patients and QRS widening occurs in 5–25% of patients [7], left bundle branch block in 4%, right bundle branch block in 3%, and nonspecific intraventricular conduction delay in 12% of patients [5]. Severe atrioventricular and intraventricular conduction defect is related to CTG repeat length and the presence of abnormal late potential (caused by slowed and fragmented conduction through damaged areas of myocardium) is directly correlated to CTG expansion and represents a substrate for malignant reentrant ventricular arrhythmias [7].

DM1, and possibly DM2, is associated with a significantly increased risk of cardiomyopathy, heart failure, conduction disorders, and arrhythmias [8]. The symptomatic presentations include palpitations, presyncope and syncope, heart failure symptoms, and sudden cardiac death [9]. Structural heart disease is also frequently observed in DM, with LV dilatation or hypertrophy observed in 20% of patients, LV systolic dysfunction in 14%, and clinical heart failure in 2% of DM1 patients based upon clinical history [10].

A 12-lead EKG is an appropriate screening test and should be performed annually after the diagnosis of DM [11]. Radionucleotide imaging and echocardiography may reveal diastolic and systolic dysfunction in either ventricle [5]. Electrophysiological study (EPS) correlating the H-V interval measurement with the electrocardiographic findings may identify predictive risk factors [12], strongly recommended in patients with clinical manifestations suggestive of ventricular tachycardia and/or with a family history of sudden death [13, 14]. In an innovative study on DM1 and cardiac disease, VT could be induced at EPS in 18% of patients in the absence of ventricular arrhythmias during Holter monitoring [14]. Among DM1 patients with major infranodal conduction delays, institution of an invasive strategy utilizing systematic electrophysiological studies with subsequent prophylactic permanent pacing indicated by malignant arrhythmias is associated with nine-year survival of almost seventy-six percent [15]. An implantable loop recorder is useful in detecting fifty percent of arrhythmias in DM1 patients and should be instituted more often in apparently asymptomatic [16] as well as high risk myotonic dystrophy patients to identify asymptomatic arrhythmias [17] which aids in the determination about antiarrhythmic devices. Cardiovascular magnetic resonance may help define the LV abnormalities of the disease including dilatation, systolic dysfunction, hypertrophy, and, occasionally, noncompaction [18, 19].

Based on the recommendation of the ACC/AHA/HRS 2008 Guidelines for device-based therapy, our patient qualified for the class IIB indication for pacemaker insertion which encompasses any degree of AV block (including first degree AV block), bifascicular block, or any fascicular block with or without symptoms (level of evidence: B) [20].

Patients with DM have an annual mortality of approximately 3.5%, one-third of which is sudden cardiac death and the systematic identification of conduction disease, and aggressive use of prophylactic pacemakers is associated with low rate of sudden cardiac death at 1.16% per year [21]. DM1 patients even when asymptomatic presenting with the Groh's criteria (prolonged PR of equal or >240 ms, wide QRS complex of equal or >120 ms, or atrial tachyarrhythmias) were at higher risk of sudden death when compared to those with normal ECGs [22]. The prophylactic implantation of pacemaker in high risk MD patients according to Groh's criteria reduced the incidence rate of sudden death to 0.2 per 100 patient-years [23].

The paroxysmal nature and the high prevalence of arrhythmias in DM patients reiterates the importance of follow-up not solely through regular ECG, but also through 24 h Holter monitoring since 32% of the patients showed conduction disturbances in the 24 h Holter monitoring that was not identified on ECG [24]. A high prevalence and changes between baseline ECG and follow-up Holter monitoring justified permanent pacemaker implantation in thirty percent of DM1 patients [25]. Current approach is to obtain 12-lead electrocardiogram each year and to consider prophylactic pacing in patients with more advanced conduction disturbances such as right bundle branch block associated with left fascicular block or bundle branch block with significant increase in PR interval, especially if there is evidence of worsening conduction over time [26]. Prophylactically inserted pacemaker in DM1 patients with HV interval of ≥70 ms even in the absence of related symptoms was monitored after implantation and was found out to have paroxysmal arrhythmias in 83.7% consisting of complete AV block, sinoatrial block, or atrial or ventricular tachyarrhythmias; thus a pacemaker including detailed diagnostic functions facilitates the diagnosis and management of frequent paroxysmal tachyarrhythmias that may remain obscure during conventional clinical surveillance [27]. Heart failure with a documented left ventricular ejection fraction of less than 50 percent should be treated with current medical treatment including angiotensin-converting enzyme inhibitors, angiotensin II receptor blockers, beta blockers, aldosterone-antagonists, and diuretics [9].

## 4. Conclusion

The timing of pacemaker implantation in Steinert's disease (DM1) should be individualized and tailored from the patient's presentation considering in the background the current clinical practice guideline's recommendation.

## References

[1] Mann D. L., Zipes D. P., Libby P., Bonow R. O., Braunwald E., Groh W. J., Zipes D. P. (2015). Neurologic disorders and cardiovascular disease. *Braunwald's Heart Disease: A Textbook of Cardiovascular Medicine*.

[2] Pelargonio G., Dello Russo A., Sanna T., De Martino G., Bellocci F. (2002). Myotonic dystrophy and the heart. *Heart*.

[3] Verhaert D., Richards K., Rafael-Fortney J. A., Raman S. V. (2011). Cardiac involvement in patients with muscular dystrophies magnetic resonance imaging phenotype and genotypic considerations. *Circulation: Cardiovascular Imaging*.

[4] Schoser B. G. H., Kress W., Walter M. C., Halliger-Keller B., Lochmüller H., Ricker K. (2004). Homozygosity for CCTG mutation in myotonic dystrophy type 2. *Brain*.

[5] McNally E. M., Sparano D. (2011). Mechanisms and management of the heart in myotonic dystrophy. *Heart*.

[6] Nguyen H. H., Wolfe J. T., Holmes D. R., Edwards W. D. (1988). Pathology of the cardiac conduction system in myotonic dystrophy: a study of 12 cases. *Journal of the American College of Cardiology*.

[7] Melacini P., Villanova C., Menegazzo E. (1995). Correlation between cardiac involvement and CTG trinucleotide repeat length in myotonic dystrophy. *Journal of the American College of Cardiology*.

[8] Lund M., Diaz L. J., Ranthe M. F. (2014). Cardiac involvement in myotonic dystrophy: a nationwide cohort study. *European Heart Journal*.

[9] Petri H., Vissing J., Witting N., Bundgaard H., Kober L. (2012). Cardiac manifestations of myotonic dystrophy type 1. *International Journal of Cardiology*.

[10] Bhakta D., Lowe M. R., Groh W. J. (2004). Prevalence of structural cardiac abnormalities in patients with myotonic dystrophy type I. *American Heart Journal*.

[11] Khalighi K., Kodali A., Thapamagar S. B., Walker S. R. (2015). Cardiac involvement in myotonic dystrophy. *Journal of Community Hospital Internal Medicine Perspectives*.

[12] Nishioka S. A. D., Filho M. M., Marie S., Zatz M., Costa R. (2005). Myotonic dystrophy and heart disease. Behavior of arrhythmic events and conduction disturbances. *Arquivos Brasileiros de Cardiologia*.

[13] Clarke N. R. A., Kelion A. D., Nixon J., Hilton-Jones D., Forfar J. C. (2001). Does cytosine-thymine-guanine (CTG) expansion size predict cardiac events and electrocardiographic progression in myotonic dystrophy?. *Heart*.

[14] Lazarus A., Varin J., Ounnoughene Z. (1999). Relationships among electrophysiological findings and clinical status, heart function, and extent of DNA mutation in myotonic dystrophy. *Circulation*.

[15] Wahbi K., Meune C., Porcher R. (2012). Electrophysiological study with prophylactic pacing and survival in adults with myotonic dystrophy and conduction system disease. *The Journal of the American Medical Association*.

[16] Stöllberger C., Steger C., Gabriel P., Finsterer J. (2011). Implantable loop recorders in myotonic dystrophy 1. *International Journal of Cardiology*.

[17] Hadian D., Lowe M. R., Scott L. R., Groh W. J. (2002). Use of an insertable loop recorder in a myotonic dystrophy patient. *Journal of Cardiovascular Electrophysiology*.

[18] Finsterer J., Stöllberger C., Kopsa W. (2005). Noncompaction in myotonic dystrophy type 1 on cardiac MRI. *Cardiology*.

[19] Ashford M. W., Liu W., Lin S. J. (2005). Occult cardiac contractile dysfunction in dystrophin-deficient children revealed by cardiac magnetic resonance strain imaging. *Circulation*.

[20] Epstein A. E., DiMarco J. P., Ellenbogen K. A. (2008). ACC/AHA/HRS 2008 guidelines for device-based therapy of cardiac rhythm abnormalities: executive summary: a report of the American College of Cardiology/American Heart Association task force on practice guidelines (writing committee to revise the ACC/AHA/NASPE 2002 guideline update for implantation of cardiac pacemakers and antiarrhythmia devices) developed in collaboration with the American Association for Thoracic Surgery and Society of Thoracic Surgeons. *Circulation*.

[21] Ha A. H., Tarnopolsky M. A., Bergstra T. G., Nair G. M., Al-Qubbany A., Healey J. S. (2012). Predictors of atrio-ventricular conduction disease, long-term outcomes in patients with myotonic dystrophy types I and II. *Pacing and Clinical Electrophysiology*.

[22] Groh W. J., Groh M. R., Saha C. (2008). Electrocardiographic abnormalities and sudden death in myotonic dystrophy type 1. *The New England Journal of Medicine*.

[23] Laurent V., Pellieux S., Corcia P. (2011). Mortality in myotonic dystrophy patients in the area of prophylactic pacing devices. *International Journal of Cardiology*.

[24] Merlevede K., Vermander D., Theys P., Legius E., Ector H., Robberecht W. (2002). Cardiac involvement and CTG expansion in myotonic dystrophy. *Journal of Neurology*.

[25] Sá M. I., Cabral S., Costa P. D., Coelho T., Freitas M., Gomes J. L. (2007). Ambulatory electrocardiographic monitoring in type 1 myotonic dystrophy. *Revista Portuguesa de Cardiologia*.

[26] Breton R., Mathieu J. (2009). Usefulness of clinical and electrocardiographic data for predicting adverse cardiac events in patients with myotonic dystrophy. *Canadian Journal of Cardiology*.

[27] Lazarus A., Varin J., Babuty D., Anselme F. R., Coste J., Duboc D. (2002). Long-term follow-up of arrhythmias in patients with myotonic dystrophy treated by pacing: a multicenter diagnostic pacemaker study. *Journal of the American College of Cardiology*.

